# Construction and Validation of a Protein Prognostic Model for Lung Squamous Cell Carcinoma

**DOI:** 10.7150/ijms.47224

**Published:** 2020-09-23

**Authors:** Xisheng Fang, Xia Liu, Chengyin Weng, Yong Wu, Baoxiu Li, Haibo Mao, Mingmei Guan, Lin Lu, Guolong Liu

**Affiliations:** 1 Department of Medical Oncology, Guangzhou First People's Hospital, School of Medicine, South China University of Technology, Guangzhou, Guangdong, China, 510180; 2 Department of Medical Oncology, Guangzhou First People's Hospital, Guangzhou Medical University, Guangzhou, Guangdong, China, 510180

**Keywords:** Lung squamous cell carcinoma, The Cancer Protein Atlas, The Cancer Genome Atlas, Protein prognostic risk model, Overall survival

## Abstract

Lung squamous cell carcinoma (LUSCC), as the major type of lung cancer, has high morbidity and mortality rates. The prognostic markers for LUSCC are much fewer than lung adenocarcinoma. Besides, protein biomarkers have advantages of economy, accuracy and stability. The aim of this study was to construct a protein prognostic model for LUSCC. The protein expression data of LUSCC were downloaded from The Cancer Protein Atlas (TCPA) database. Clinical data of LUSCC patients were downloaded from The Cancer Genome Atlas (TCGA) database. A total of 237 proteins were identified from 325 cases of LUSCC patients based on the TCPA and TCGA database. According to Kaplan-Meier survival analysis, univariate and multivariate Cox analysis, a prognostic prediction model was established which was consisted of 6 proteins (CHK1_pS345, CHK2, IRS1, PAXILLIN, BRCA2 and BRAF_pS445). After calculating the risk values of each patient according to the coefficient of each protein in the risk model, the LUSCC patients were divided into high risk group and low risk group. The survival analysis demonstrated that there was significant difference between these two groups (p= 4.877e-05). The area under the curve (AUC) value of the receiver operating characteristic (ROC) curve was 0.699, which suggesting that the prognostic risk model could effectively predict the survival of LUSCC patients. Univariate and multivariate analysis indicated that this prognostic model could be used as independent prognosis factors for LUSCC patients. Proteins co-expression analysis showed that there were 21 proteins co-expressed with the proteins in the risk model. In conclusion, our study constructed a protein prognostic model, which could effectively predict the prognosis of LUSCC patients.

## Introduction

Lung cancer is the most common and the leading cause of cancer-related malignancy in the world 1. According to the Cancer statistics 2018, there were about 2.09 million new cases and 1.76 million deaths per year 2. Despite advancements in chemotherapy, radiotherapy, targeted therapy, biotherapy and immunotherapy, the 5-year survival rate of LUSCC is only 18% 3-5. And no individualized treatment strategy is recommended to LUSCC due to the lack of clear histological features 6. Besides, the economic impact of LUSCC is significant and is increasing yearly. Therefore, it is urgent and necessary to find more specific and reliable diagnostic and prognostic biomarkers for LUSCC.

Several studies have suggested prognostic factors for LUSCC, including proteins, mRNAs, lncRNAs and miRNAs. Tumor protein biomarkers demonstrated important roles in cancer detection, clinical outcome prediction or effective therapy selection 7-9. Of note, protein biomarkers are economical, reliable and easily measurable. For instance, carcinoembryonic antigen (CEA), as the only FDA-approved biomarker for colorectal cancer (CRC), is a selective biomarker for detecting disease recurrence and monitoring response to routine treatment 10, 11. Besides, proteins, such as tripartite motif-containing protein59 (TRIM59), minichromosome maintenance protein 2 (MCM2) and CD271 were highly expressed, while the expression of interferon-induced proteins with tetratricopeptide repeats 2 (IFIT2) was decreased in LUSCC cell lines or tissues 12. The overall survival (OS) of patients with high TRIM59, MCM2, CD271 expression or low IFIT2 expression were significantly better than the opposite group 12-16. However, the sensitivity and specificity was not satisfactory using one biomarker. Prognostic model using multiple protein biomarkers would demonstrate great potential in the diagnosis and prognosis of LUSCC patients.

In this study, we presented a prognostic risk model which was consisted of 6 proteins by identifying 237 proteins from TCPA database and TCGA database. In addition, we determined that the risk model was an independent prognostic model for LUSCC patients. This novel prognostic risk model could provide evidence for the diagnosis and prognosis of LUSCC patients.

## Material and methods

### Protein data download and data processing

Protein expression data of LUSCC patients were downloaded from The Cancer Proteome Atlas (TCPA) database up to January 2020. The “impute” package in the R software was used to fill up the missing data. This study complied with the publication guidelines for TCPA. And no additional ethical consent was required.

### Clinical data download and data consolidation

Clinical data of LUSCC patients were downloaded from the TCGA database up to January 2020. Perl software was used to extract survival information and merge it with the protein expression data. This study complied with the publication guidelines of TCGA. And no additional ethical consent was required.

### Kaplan-Meier survival analysis and univariate Cox analysis

The correlations between protein expression and overall survival of LUSCC patients were analyzed using Kaplan-Meier survival analysis and univariate Cox analysis via “survival” package in R software. A *P* value < 0.05 was used as the criterion for screening proteins with significant difference. The "ggplot2" and "ggrepel" packages were used to draw the volcano map of differently expressed proteins.

### Construction of a prognostic risk model

The proteins with statistical difference were analyzed by multivariate Cox analysis to build a prognostic risk model. The coefficients of each protein in the model and the risk values of all samples were obtained at the same time. According to the risk values, patients were divided into high risk group and low risk group.

### Assessment of the performance of the prognostic risk model

The "survminer” and “survival” package in R software were used to analyze the relationships between component proteins, risk values and survival status, as well as the survival curve. Using the "pheatmap" package in the R software, we built a risk curve for risk value, survival status and protein expression. The survival time, survival state, risk value, age, gender, staging, T, M and N states of the samples were combined with Perl software, followed by univariate and multivariate COX analysis to observe the correlations between the above clinical states, risk value and survival state. The ROC curve with the AUC value were assessed by the "survivalROC" package in R software.

### Co-expressed proteins analysis

Other proteins co-expressed with the proteins in the risk model were screened by R software with the correlation coefficient was set by > 0.5 and the *P* value was set by < 0.001. The packages of "ggalluvial, ggplot2 and dplyr" in R software were used to construct the sankyl diagram for the related co-expressed proteins.

### Statistical analysis

The retrieval of protein data and clinical information from TCPA and TCGA dataset were performed in R software (R 3.6.2) or Perl software Strawberry Perl (64-bit). All statistical analyses were assessed by R software. A *P*-value < 0.05 was statistically significant.

## Results

### Protein and clinical data download and data processing

**Figure 1:**
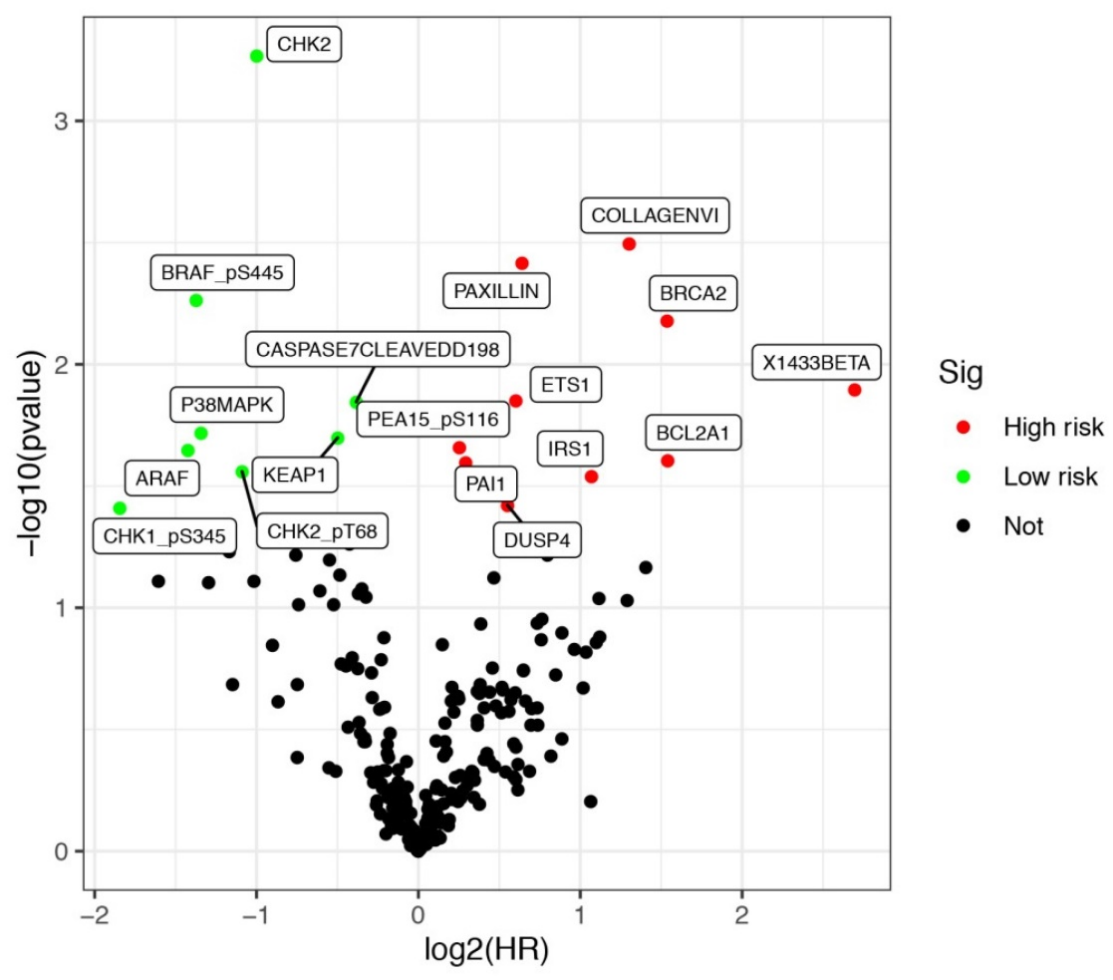
** Differentially expressed proteins in lung squamous cell carcinoma (LUSCC).** Data were retrieved from TCPA and TCGA database. A total of 320 cases of LUSCC patients with both protein expression data and clinical parameters were included.

Protein expression data of LUSCC patients were downloaded from the TCPA database up to January 2020. The database contains 8167 tumor samples in total. And it is mainly consisted of TCGA tumor tissue sample sets, which including more than 32 cancer types in TCGA, and about 500 samples from an independent patient cohort. Bisides, The database also focuses on reverse-phase protein arrays (RPPA) data for cancer cell lines, containing >665 individual cell lines across 19 lineages (https://tcpaportal.org/tcpa/index.html). A total of 237 proteins were identified from 325 cases of LUSCC patients. Meanwhile, clinical data of 504 cases of LUSCC patients were downloaded from TCGA database. After screening 320 cases with both survival information and protein expression data, a total of 17 proteins were identified significantly associated with the survival of LUSCC patients. All the differentially expressed proteins was demonstrated by volcano plots (Figure 1). Among them, COLLAGENVI, PAXILLIN, BRCA2, X1433BETA, ETS1, PEA15_pS116, BCL2A1, PAI1, IRS1 and DUSP4 were regarded as high risk factors, while BRAF_pS445, CASPASE7CLEAVEDD198, P38MAPK, KEAP1, ARAF, CHK2_pT68 and CHK1_pS345 were viewed as low risk factors.

### Kaplan-Meier survival analysis and univariate Cox analysis

**Figure 2:**
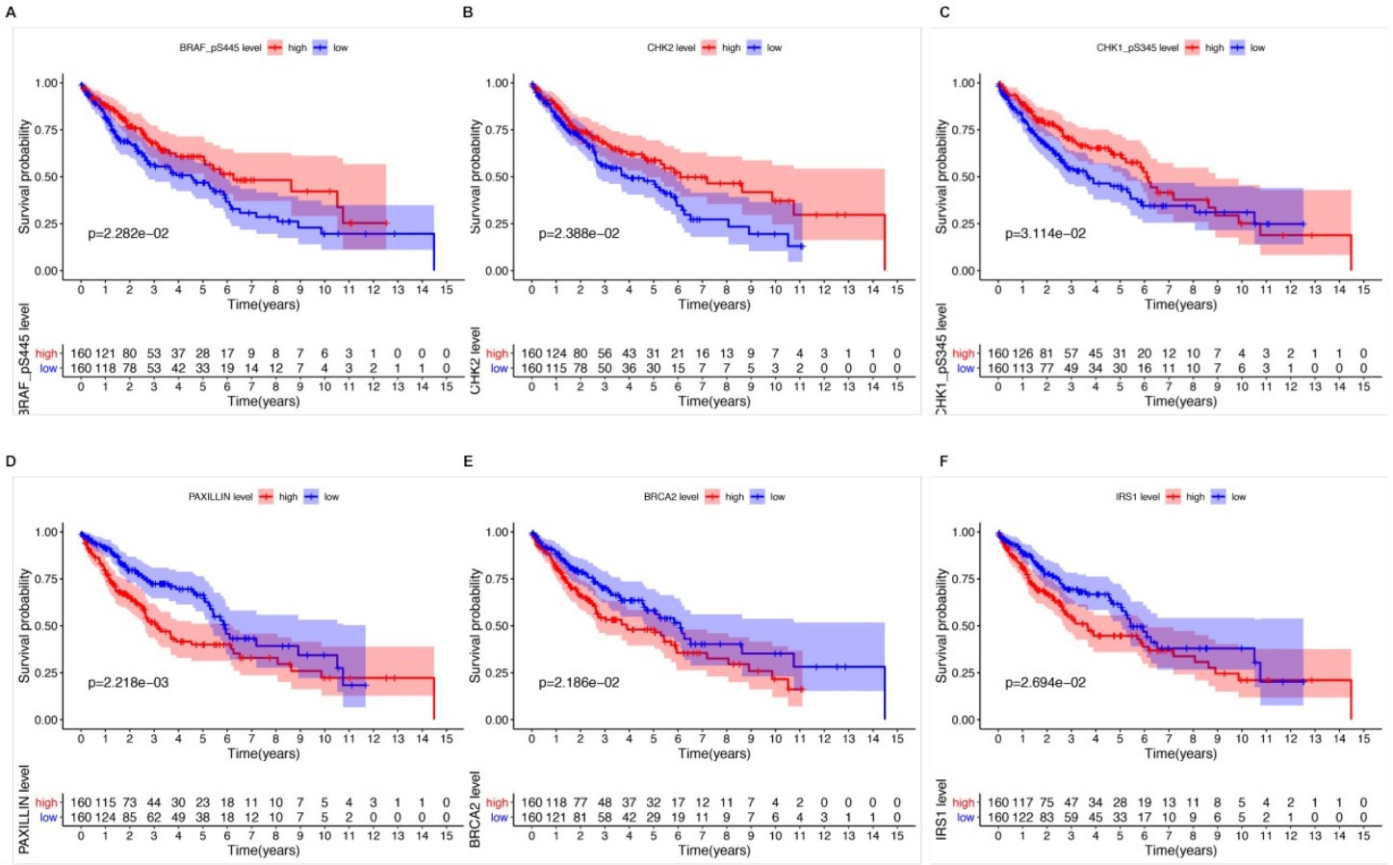
** The relationships between six independent prognostic proteins and overall survival (OS) of LUSCC patients.** High expression of CHK1_pS345 (A), CHK2 (B), BRAF_pS445 (C) were positively correlated with better OS. High expression of PAXILLIN (D), BRCA2 (E) and IRS1(F) indicated poor OS.

Kaplan-Meier survival analysis and univariate Cox analysis demonstrated that 6 proteins were significantly correlated with OS and could be viewed as independent prognostic factors for LUSCC patients at a criteria of P value < 0.05 (Table 1). High expression of CHK1_pS345 (P=3.114e-02, Figure 2A), CHK2 (P=2.388e-02, Figure 2B), BRAF_pS445 (P=2.282e-02, Figure 2C) were positively correlated with better overall survival. While high expression of PAXILLIN (P=2.218e-03, Figure 2D), BRCA2 (P=2.186e-02, Figure 2E) and IRS1 (P=2.694e-02, Figure 2F) indicated poorer OS for LUSCC patients.

### Construction of a prognostic risk model

**Figure 3:**
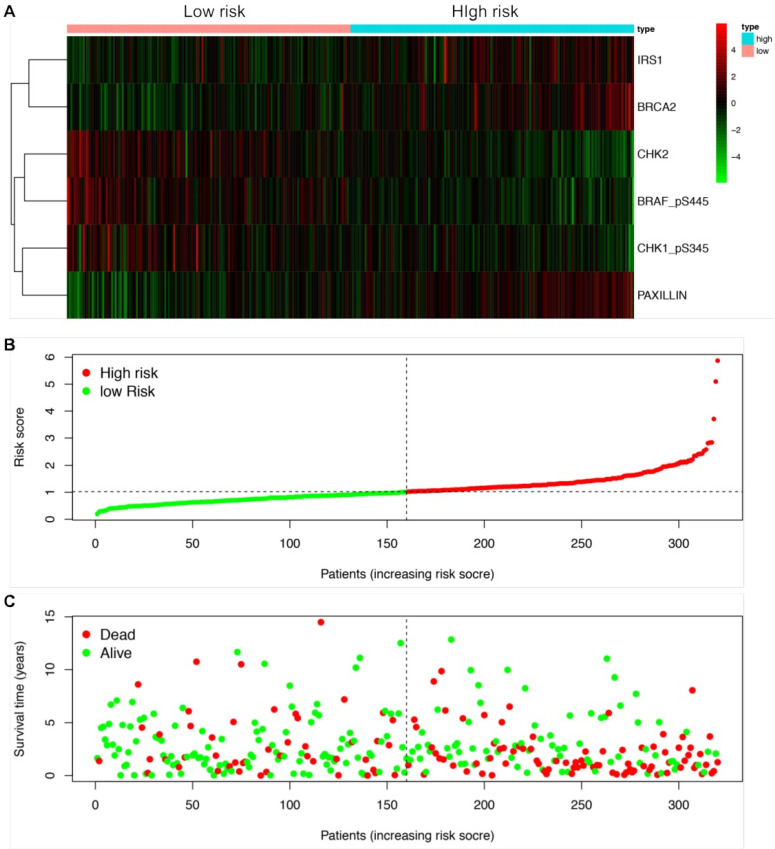
** Construction of a protein prognostic risk model in LUSCC.** The patients were divided into high-risk group and low-risk group according to the risk values. (A) The heatmap demonstrated the expression of the six proteins between high risk group and low risk group. Upregulated expression of CHK1_pS345, CHK2 and BRAF_pS445 were detected in low-risk group, while upregulated expression of PAXILLIN, BRCA2 and IRS1 were detected in high-risk group. (B) Scatter diagram shows the distributions of risk scores of LUSCC patients. (C) Scatter diagram shows the survival status of the patients based on this prognostic risk model.

We next established a prognostic risk model using multivariate Cox regression analysis. According to the multivariate Cox analysis, a total of 6 proteins were included in the prognostic risk model, which could be viewed as independent prognostic factors for LUSCC patients (Table 2). The coefficients of each protein were demonstrated in Table 2. After calculating the risk value of each patient according to the coefficient of each protein in the risk model, the LUSCC patients were divided into high risk group and low risk group. Each group contained 160 cases. The heatmap shows the protein expression profiles of the prognostic risk model based on the risk scores (Figure 3A). Figure 3B shows the risk score distribution of the LUSCC patients. Figure 3C shows the survival status of the LUSCC patients based on the risk scoring model.

### Assessment of the performance of the prognostic risk model

**Figure 4:**
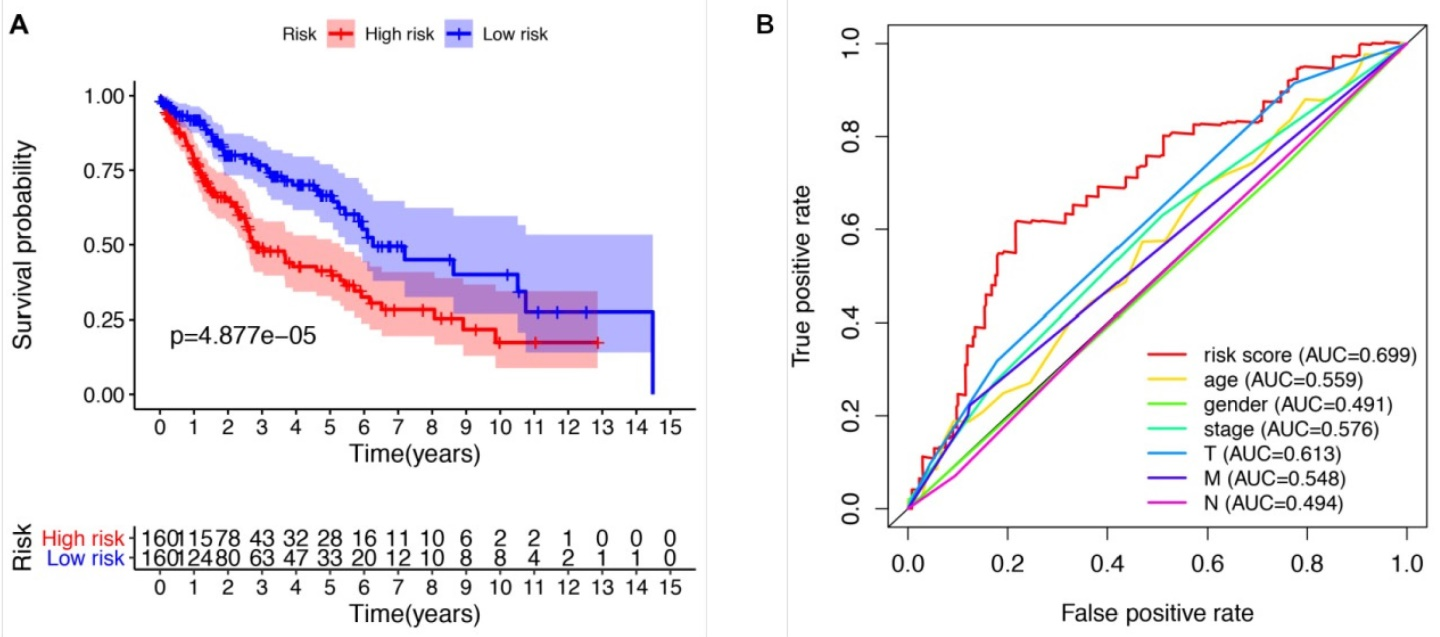
** The prognostic risk model could effectively predict the survival of LUSCC patients.** (A) LUSCC patients in high-risk group demonstrated poor OS than that in the low-risk group. (B) Receiver operating characteristic (ROC) curve revealed the performance of the prognostic risk model in LUSCC.

**Figure 5:**
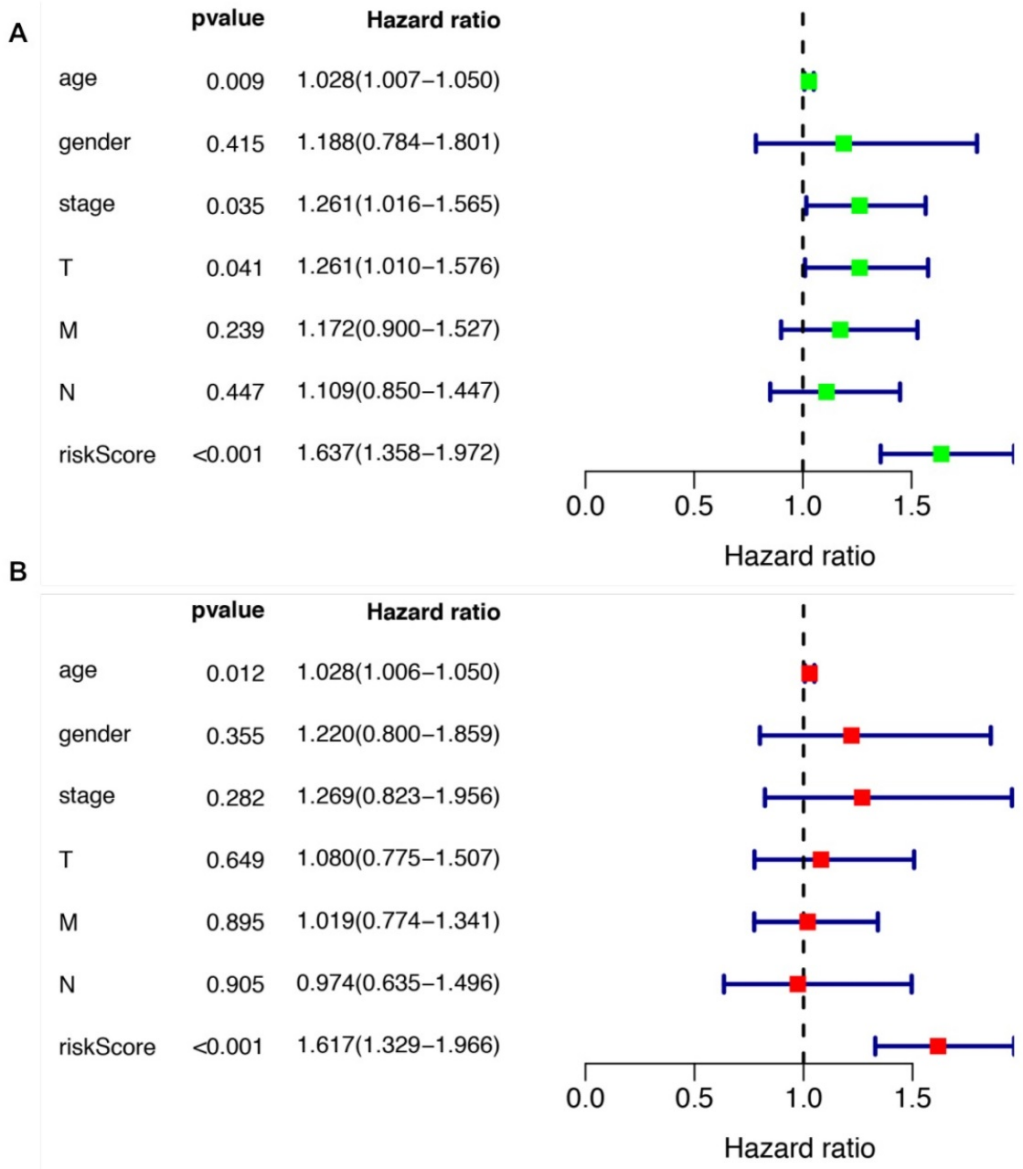
** The prognostic significances of clinicopathological parameters and the risk model.** (A) Univariate Cox analysis was performed to assess the prognostic values of various clinicopathological factors and risk score. (B) Multivariate Cox analysis revealed the independent prognostic values of various clinicopathological factors and risk score in the survival of LUSCC patients.

We further evaluated the performance of the prognostic risk model. After being divided into two groups based on the risk scores, there was significant difference between high risk group and low risk group in the OS (*P* = 4.877e-05, Figure 4A). As shown in Figure 4B, the area under the curve (AUC) value was 0.699 in the receiver operating characteristic (ROC) curve of the risk model, which suggesting that the prognostic model can effectively predict the survival of LUSCC patients. Moreover, combining the risk value with survival time, survival state, age, gender, stage, T, M and N states with Perl software, univariate Cox analysis demonstrated that the risk value (HR 1.673(1.358-1.972), P<0.001), age (HR 1.028(1.007-1.050), P=0.009), stage (HR 1.261(1.016-1.565), P=0.035), T states (HR 1.261(1.010-1.576), P=0.041) were statistical significance (Figure 5A). Further multivariate Cox analysis revealed that the risk value (HR 1.617, 95%CI 1.329-1.966, *P*=1.51e-06) and age (HR 1.028, 95%CI 1.006-1.050, *P*=0.012) could be viewed as independent prognostic factors for LUSCC patients (Figure 5B).

### Co-expressed protein analysis

**Figure 6:**
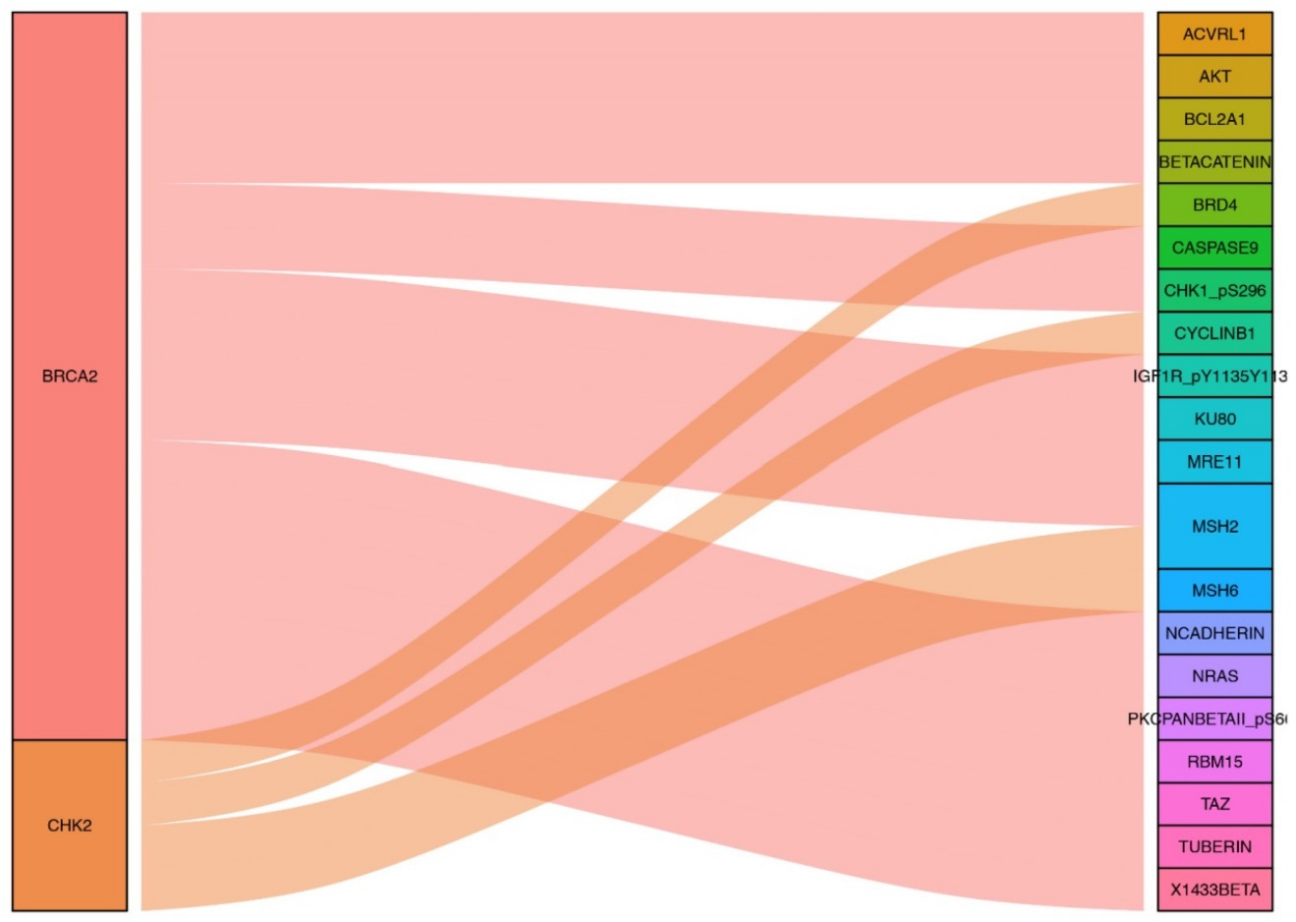
Sankyl diagram shows other proteins co-expressed the six proteins included in the prognostic risk model.

Other proteins co-expressed with the proteins included in the prognostic model were screened by R software with the correlation coefficient was set by > 0.5 and the *P* value was set by < 0.001. AKT, BETACATENIN, KU80, MRE11, NCADHERIN, TUBERIN, NRAS, PKCPANBETAII_pS660, RBM15, TAZ, X1433BETA, ACVRL1, MSH2, BCL2A1, CHK1_pS296, IGF1R_pY1135Y1136 and CASPASE9 were found to be co-expressed with BRCA2. And proteins co-expressed with CHK2 were CYCLINB1, MSH2, MSH6 and BRD4. The sankyl diagram for the related co-expressed proteins was constructed by packages of “ggalluvial, ggplot2 and dplyr” in R software (Figure 6).

## Discussion

With the development of next-generation sequencing (NGS) technologies, there were more and more risk factors being identified for LUSCC patients, including miRNAs, lncRNAs and mRNAs 17-20. However, some disadvantages of NGS, such as cost, reproducibility, and data analysis, had widely limited its application. Moreover, tumor protein biomarker was economical, reliable and clinical feasible 7, 21. Besides, it would be more accessible in health-providing centers. Some studies had successfully validated protein prognostic model in malignancies, such as bladder cancer, lung adenocarcinoma and diffuse large B-cell lymphoma 22-24. The protein models demonstrated vital role for predicting prognosis, which including bladder cancer, esophageal squamous cell carcinoma and pancreatic ductal adenocarcinoma 25-27. Abnormal expression of various protein biomarkers in cancer cells or inflammatory cells demonstrated prediction potential in cancers 21, 28, 29. And a prediction model would demonstrate much better specificity and sensitivity than one biomarker.

In this study, a total of 320 cases of LUSCC patients with both clinical information and protein expression data were identified from the TCPA and TCGA database. We further established a prognostic risk model that including 6 proteins (CHK1_pS345, CHK2, IRS1, PAXILLIN, BRCA2 and BRAF_pS445) using multivariate Cox regression analysis. These six proteins were significantly correlated with OS of LUSCC patients. In addition, the performance of the prognostic risk model was evaluated according to Kaplan-Meier survival analysis and ROC curve. Moreover, we screened other proteins co-expressed with the proteins in the risk model to find more related proteins. Our study validated a prognostic risk model which was with good sensitivity and specificity, and could effectively predict the survival of LUSCC patients.

Some of the proteins in our prognostic risk model have been reported to be involved in the tumorigenesis and prognosis of malignancies. Check-point kinase (Chk) is an enzyme that plays multiple roles in cancer development, including (1) blocking cell cycle and DNA damage, (2) mediating the detection and repair of damaged DNA, (3) guaranteeing the quality of DNA replication and distribution of chromosome^30^. Chk1 and Chk2 were the most common enzymes in ChK family, both played extremely important roles in DNA damage repair^31^. The deletion or overexpression of Chk1 or Chk2 may cause the occurrence and development of some tumors and affect the prognosis of cancer patients 32-34. Insulin Receptor Substrate (IRS) proteins are the main cytoplasmic adaptor molecules involving in transducing extracellular signals from receptors to downstream proteins. IRS family is consisted of four proteins (IRS1-IRS4). IRS1 is located on human chromosome 2q34-37 and encoded by single exon. It is widely expressed in human tissues 35. IRS1 expression was over-expressed in laryngeal squamous cell carcinoma and colorectal cancer 36, 37. Using single-cell analysis, Eyler et al. found that glioma cells could activate the AKT signaling pathway through IRS1 to acquire drug resistance capability 38. Furthermore, IRS1 Gly972Arg variations may be associated with an increased susceptibility to develop gastric cancer 39.The important cellular adhesion factor Paxillin (PXN) is a hallmark protein in focal adhesion (FA) site. It acted as a signal binding protein of tumorigenic tyrosine kinases and was involved in various physiological processes, such as cell proliferation, tissue reconstruction, cell adhesion and migration 40. High expression of PXN was correlated with worse prognosis in liver and colorectal cancer 41-43. Various researches have suggested that breast cancer susceptibility gene 2 (BRCA2) was widely involved in gene transcription and cell apoptosis 44, 45. Mutation of BRCA2 genes led to function loss of BRCA2 protein, which resulted in tumorigenesis, progression and poor prognosis 46-48. BRAF pS445 was an essential kinase in the mitogen-activated protein kinase (MAPK) pathway. Mutation of BRAF gene led to uncontrollable cell growth and ultimately developed to cancer cell 49. Besides, application of BRAF inhibitors could improve outcomes of melanoma, colorectal cancer and non-small cell lung cancer patients 50-52.

In addition, some studies have revealed the expression and biological functions of these proteins in lung cancer. CHK1 was upregulated in lung cancer cells. Inhibition of CHK1 could seriously damage the cell cycle characteristics of lung cancer cells 53. Activation of Chk1 was the earliest and most important event in NSCLC stem cells receiving chemotherapy. Differentiated NSCLC cells were usually accompanied with weak Chk1 activation and more sensitive to chemotherapeutic agents than undifferentiated tumor cells. In vitro, chemotherapy combined with Chk1 inhibitor can significantly reduce the survival rate of NSCLC stem cell 54. Targeting Chk2 reduced phosphorylation and increased the efficacy of cisplatin 55. Researches showed that genomic changes of IRS1 could lead to development of lung cancer 56. Silencing IRS1 caused lung cancer cell proliferation and induced phosphorylation of anaplastic lymphoma kinase(AKT) 57. Importantly, Paxillin played a crucial role in the progression of lung cancer 58. Studies by Jagadeeswaran et al. 59 identified a total of 21 unique paxillin mutations in lung cancer specimens and cell lines. BRCA2 gene has been proved to carry a high risk of hereditary in breast and ovarian cancer. A stop-gain mutation of this gene, K3326* (rs11571833), was significantly associated with small cell lung cancer 60. Besides, activating BRCA2 by MIR-1245 suppressed the proliferation and invasion of lung cancer cells 61. The polymorphism rs144848 in gene BRCA2 often suggested a lower incidence of lung cancer in Chinese women 62. BRAF mutations were detected in 3% of NSCLC, in which 58% was BRAF V600E mutation and the mutations were always associated with smoking 63, 64. Recent reports revealed that it acted as resistance pathways in 3-10% of osimertinib-treated patients 65-68. To targeting BRAF mutations, vemurafenib was an option^69^. Another therapeutic approach such as ulixertinib worked by blocking downstream signaling in the MAP kinase pathway^70^. However, there are lack of studies concerning these protein biomarkers in LUSCC. Thus, the expression and biological functions of these proteins in LUSCC are needed to be further elucidated in our future work.

In conclusion, we have established and validated a protein-based prognostic risk model for LUSCC patients. This model demonstrated good sensitively and specificity. Integrated analysis of proteins would provide economic, accuracy and stability pattern in research of diagnosis and prognosis biomarkers for LUSCC patients.

## Figures and Tables

**Table 1:** Significant proteins from Kaplan-Meier and univariate COX analysis (*P* < 0.05)

protein	Kaplan-Meier	HR	HR.95L	HR.95H	P value
**CHK1_pS345**	0.031	0.278	0.0826	0.937	0.039
**CHK2**	0.024	0.500	0.338	0.741	0.001
**IRS1**	0.027	2.100	1.079	4.088	0.029
**PAXILLIN**	0.002	1.560	1.154	2.108	0.004
**BRCA2**	0.022	2.905	1.345	6.273	0.007
**BRAF_pS445**	0.023	0.387	0.198	0.756	0.005

**Table 2:** Significant proteins from multivariate Cox analysis

protein	coef	HR	HR.95L	HR.95H	P value
**CHK1_pS345**	-0.982	0.375	0.093	1.503	0.166
**CHK2**	-0.324	0.723	0.454	1.153	0.173
**IRS1**	-0.077	0.926	0.399	2.147	0.858
**PAXILLIN**	0.443	1.557	1.132	2.140	0.006
**BRCA2**	0.563	1.755	0.600	5.135	0.304
**BRAF_pS445**	-0.706	0.494	0.249	0.979	0.043

## Abbreviations

LUSCC: Lung squamous cell carcinoma
TCPA: The Cancer Protein Atlas
TCGA: The Cancer Genome Atlas
ROC: receiver operating characteristic
AUC: area under the curve
CEA: carcinoembryonic antigen
CRC: colorectal cancer
TRIM59: tripartite motif-containing protein59
MCM2: minichromosome maintenance protein 2
IFIT2: interferon-induced proteins with tetratricopeptide repeats 2
OS: overall survival
NGS: next-generation sequencing
Chk: Check-point kinase
IRS: Insulin receptor substrate
PXN: Paxillin
FA: focal adhesion
MAPK: mitogen-activated protein kinase
AKT: Anaplastic lymphoma kinase
